# A Vascular–Extracellular Matrix Molecular Program Identifies High-Risk Diffuse Glioma Across Independent Multi-Omics

**DOI:** 10.3390/cancers18101652

**Published:** 2026-05-20

**Authors:** Shamsa Hilal Saleh, Arshiya Akbar, Fareeha Arshad, Saniyah Shaikh, Volodymyr Mavrych, Olena Bolgova, Abrar Barakzai, Ahmed Abu-Zaid, Mohammed Imran Khan, Itika Arora, Ahmed Yaqinuddin

**Affiliations:** 1 College of Medicine, Alfaisal University, Riyadh 11533, Saudi Arabia; sanzi@kfshrc.edu.sa (S.H.S.); arshiyaakbar2019@gmail.com (A.A.); farshad@alfaisal.edu (F.A.); sanshaikh@alfaisal.edu (S.S.); vmavrych@alfaisal.edu (V.M.); obolgova@alfaisal.edu (O.B.); abarakzai@alfaisal.edu (A.B.); amabuzaid@alfaisal.edu (A.A.-Z.); mikhan@kfshrc.edu.sa (M.I.K.); 2 King Faisal Specialist Hospital and Research Center, Jeddah 23433, Saudi Arabia; 3 Center for Biotechnology, Khalifa University of Science and Technology, Abu Dhabi 127788, United Arab Emirates

**Keywords:** diffuse glioma, glioblastoma, MOFA, multi-omics integration, tumor purity, IDH stratification, cross-cohort validation, concordance index

## Abstract

Background: Gliomas are characterized by a high degree of molecular heterogeneity, which impairs the reproducibility of predictive biomarkers derived from bulk-based molecular profiling due to immune/stromal contamination of tumors and the high prevalence of the IDH mutation signature. Methods: In this study, we used MOFA+ to derive intrinsic molecular signatures from transcriptional, methylation, and genomic profiles of a cohort of 667 diffuse gliomas in the Cancer Genome Atlas database. Thereafter, factor scores were derived for two separate Chinese Glioma Genome Atlas batches (Batch 1, *n* = 325; Batch 2, *n* = 693) without any retraining on the model. The prognostic independence of identified molecular signatures was assessed using multivariable Cox regression adjusted for IDH mutation status and tumor purity; purity-residualized survival analyses; IDH-stratified Cox regression in each cohort; validation by concordance index against established molecular signatures; and survival extreme profiling. To characterize the biological significance of factor signatures, we projected gene set signatures corresponding to each factor signature onto a single-cell RNA-seq dataset of GBM (GSE131928). Results: MOFA+ identified 12 latent factors, of which a vascular–extracellular matrix (ECM) remodeling axis (Factor 1) explained the highest multi-omics variance (24.9%) and was the strongest independent prognostic factor. In multivariable Cox regression adjusting for IDH status and tumor purity, Factor 1 remained independently prognostic (HR = 1.67, 95% CI 1.27–2.20, *p* = 0.0002); in a fully-adjusted model additionally including age, WHO grade, MGMT methylation, and 1p/19q codeletion (plus radiotherapy and chemotherapy status in the CGGA cohorts), Factor 1 remained prognostic in both CGGA cohorts (CGGA1: HR = 1.50, *p* = 3.8 × 10^−5^; CGGA2: HR = 1.18, *p* = 0.003) but lost significance in TCGA (HR = 1.04, *p* = 0.83), consistent with the cohort-dependent magnitude reported in the IDH-stratified and meta-regression analyses below. Purity-residualized survival analysis showed negligible attenuation of the Factor 1 signal (raw HR = 3.57 vs. residualized HR = 3.72; concordance 96.5%). Within IDH-wildtype gliomas, Factor 1 was significant in both external validation cohorts (CGGA1: HR = 1.64, FDR = 4.6 × 10^−6^; CGGA2: HR = 1.20, FDR = 0.02), though the TCGA IDH-wildtype subgroup showed a trend that did not survive FDR correction (FDR = 0.060). All validation was performed without model retraining. Within IDH-mutant gliomas, Factor 1 was strongly prognostic in both CGGA cohorts but was not significant in TCGA (HR = 1.17, FDR = 0.33). These findings should therefore be interpreted as consistent in directionality across cohorts but not uniformly replicated at the FDR-adjusted significance threshold in the TCGA discovery dataset. Concordance index benchmarking on a matched subset (*n* = 503) showed Factor 1 achieved discrimination comparable to the Mesenchymal signature (C = 0.797 vs. 0.801; ΔC = −0.004) while outperforming four other established classifiers. Factor 1 consistently separated patients with extreme survival phenotypes (OS < 6 vs. >15 months) across all three cohorts (all log-rank *p* < 0.001). Projection onto a single-cell GBM atlas (GSE131928), supported by inferCNV-based malignant-cell classification, localized the Vascular–ECM program to malignant cells and the Immune–ECM axis to myeloid compartments. Conclusions: The Vascular–ECM axis is a consistent, prognostic program robust to purity adjustment for diffuse gliomas that remains relevant across IDH-defined subgroups in three independent datasets comprising 1685 patients. The Vascular–ECM axis is a reproducible, purity-robust prognostic program in diffuse glioma, with directionally consistent adverse effects across TCGA, CGGA Batch 1, and CGGA Batch 2 (pooled *n* = 1685). Given the strong co-loading of endothelial, ECM, and myeloid genes observed in the single-cell projection, Factor 1 is best interpreted as a vascular/ECM-associated tumor–microenvironment ecosystem program rather than a malignant-cell-autonomous signature. Its FDR-adjusted significance within IDH-stratified subgroups is cohort-dependent and robust in both CGGA cohorts but attenuated in the TCGA IDH-wildtype (FDR = 0.060) and TCGA IDH-mutant (FDR = 0.33) strata. The pooled signal should therefore be interpreted as evidence of a generalizable biological program rather than a uniformly replicated subgroup-specific biomarker. It is possible to calculate factor scores based on RNA sequencing alone using fixed loadings (Z = XWᵀ), which may have implications for future translational applications. All findings are correlative; a causal role for the Vascular–ECM program in glioma progression, invasion, or therapy resistance remains to be established through functional perturbation experiments.

## Simple Summary

Diffuse gliomas such as glioblastoma represent biologically heterogeneous brain tumors with few established prognostic biomarkers. One potential limitation of using bulk tumor omics to discover prognostic biomarkers is that the profiles will include both malignant and benign cells, which may obscure the underlying biology of interest. Here, we developed and implemented a purity-corrected multi-omics integration approach using data from 667 diffuse gliomas in The Cancer Genome Atlas and identified three reproducible biological axes (one predominantly malignant-cell-associated and two with strong microenvironmental contributions) (Vascular–ECM, Immune–ECM, Proliferative/cell-cycle) in these tumors. A program related to vascular and extracellular matrix remodeling accounted for the largest share of variability and had the highest association with poor prognosis among these programs. Interestingly, even after stringent correction for tumor purity and IDH mutations, the vascular and extracellular matrix remodeling program maintained its independent prognostic power. Specifically, the program remained prognostic after adjustment for both purity (−4.1% attenuation; 96.5% concordance) and IDH mutation status, and its discriminatory ability was comparable to established molecular classifiers such as the Mesenchymal signature. The biological findings were further validated by projecting them onto two independent Chinese Glioma Genome Atlas validation cohorts (*n* = 1018; pooled with TCGA, total *n* = 1685). Importantly, no retraining of the model was necessary to validate our findings, and factor scores can be derived from standard RNA-seq measurements alone.

## 1. Introduction

Diffuse gliomas encompass a spectrum of primary brain tumors with diverse molecular profiles and clinical outcomes. Among these, glioblastoma (GBM) is the most common and aggressive subtype, with a median overall survival of only ~15 months despite maximal safe resection followed by concurrent temozolomide chemoradiotherapy and adjuvant chemotherapy [1,2,3,4]. Diffuse gliomas account for approximately 80% of all malignant primary brain tumors, with an age-adjusted incidence of ~6 per 100,000 person-years for glioblastoma in the United States and a 5-year survival below 7% for IDH-wildtype GBM (CBTRUS 2020 [2]). Despite the WHO 2021 molecular reclassification (IDH, 1p/19q, MGMT, CDKN2A/B, TERT), prognostic stratification within IDH-wildtype GBM and IDH-mutant astrocytoma remains inadequate: Verhaak transcriptional subtypes are unstable across cohorts and lose power after purity correction [5,6], and the mesenchymal signature, currently the best-performing classifier, relies on discrete subtype assignment and does not capture the graded biology of vascular–ECM remodeling. An RNA-seq-only, continuous, purity-robust score that is directly portable across cohorts without retraining would therefore fill a concrete gap in clinical trial enrichment and post-surgical risk stratification. Despite a wealth of clinical and molecular data, no targeted therapy has significantly improved survival outcomes over the current standard of care, and predictive biomarkers for patient stratification in clinical trials remain elusive [5,6]. A major obstacle arises from the widespread molecular heterogeneity of GBM, encompassing not only inter-patient variability but also intra-tumoral diversity, which blurs reproducible biological signals and limits generalization across cohorts [7,8].

Recent large-scale multi-omics profiling as part of programs such as The Cancer Genome Atlas (TCGA) enables unprecedented molecular resolution across transcriptomic, epigenomic, and genomic layers [5,9]. Integrative analysis of such datasets has revealed recurrent molecular subtypes, epigenetic programs, and copy-number alterations that characterize GBM biology [10,11]. A critical and regularly underappreciated confounding factor of bulk tumor profiling, though, is tumor purity, the fraction of sequenced material that can be attributed to malignant cells vs. infiltrating immune cells, stromal fibroblasts, and vascular endothelium [12,13]. Since GBM is an uncommonly immunogenic and stroma-infiltrated tumor, the vast majority of bulk molecular signals are contaminated by microenvironmental admixture, leading to factors that merely reflect cell-type composition rather than intrinsic tumor biology [14,15,16]. These confounding factors limit the reproducibility of prognostic signatures across independent profiled cohorts under different conditions.

Multi-omics factor analysis provides a principled framework for decomposing coordinated variability across heterogeneous molecular data types into biologically interpretable latent axes [17,18]. MOFA+ (Multi-Omics Factor Analysis v2) generalizes this framework to multiple molecular views and groups in parallel, using a probabilistic generative model with automatic relevance determination to enable data-driven discovery of axes that simultaneously explain shared and view-specific variance. Crucially, by acting on continuous latent factors rather than discrete subtypes, MOFA+ can identify graded biological programs that are preserved across cohorts and can be scored with out-of-sample projection without retraining the model, a property critical to true external validation [18]. Although MOFA+ offers several methodological benefits, it has yet to be used for diffuse glioma with purity adjustment, and the consistency of MOFA+ prognostic axes defined in IDH subgroups of patients across different datasets has not been explored. A recent preprint analyzing glioma omics data using MOFA has reported distinct latent factors with prognostic relevance [19], consistent with a peer-reviewed multi-omic glioblastoma study [20].

The IDH mutation status is the most powerful molecular stratifier in diffuse glioma, clearly separating IDH-mutant lower-grade gliomas from their IDH-wildtype counterparts, including GBM, in terms of epigenetic landscape, metabolic reprogramming, and clinical outcomes [21,22,23,24,25]. Despite this, considerable prognostic heterogeneity within the IDH-wildtype GBM population remains unexplained by IDH status alone [8,10], highlighting the need for additional molecular axes that capture both malignant and microenvironmental biology. The hallmark of GBM progression is vascular remodeling and ECM reorganization, which drive invasion, angiogenesis, and therapeutic resistance [26,27]. Tumor-associated immune and microglial activation forms a second major axis of biological variability, driven by the induction of distinct myeloid and lymphoid programs that sculpt an immunosuppressive microenvironment [15,28]. Proliferative and cell-cycle programs represent another recurring axis with well-defined prognostic importance in GBM [5,11].

In this study, we conduct a purity-corrected multi-omics factor analysis on 667 diffuse gliomas from TCGA and further test the association with 1018 samples from two distinct CGGA patient groups. The study identifies a leading vascular-ECM remodeling pathway, along with other pathways, including immune-ECM activation and the proliferative/cell-cycle pathway. It assesses its prognostic value by conducting a multivariable Cox regression model adjusted for IDH mutation and tumor purity, stratifying by IDH mutation across all three data sets, comparing the concordance index with well-established molecular signatures, and phenotyping using survival extremes based on predefined clinical cutoffs. Biological anchoring of factor programs was performed via projection onto an independent GBM single-cell RNA-seq atlas. Importantly, all prognostic findings are independently validated by out-of-sample projection into two fully independent CGGA cohorts (*n* = 1018) without model retraining, establishing cross-cohort reproducibility as a primary analytical parameter. This framework provides a reproducible approach for molecular stratification of diffuse glioma, with deep implications for biomarker development and clinical trial design.

## 2. Materials and Methods

### 2.1. Study Cohorts and Data Sources

The discovery cohort included TCGA diffuse gliomas with available matched RNA-seq, DNA methylation (Illumina HumanMethylation450), and gene-level copy-number alteration (CNA) profiles. After this filtering step, only tumors with information across all three molecular layers were retained, resulting in 667 samples (Supplementary Table S1). Two independent CGGA RNA-seq cohorts were used for external validation: CGGA Batch 1 (*n* = 325; 313 with survival information) and CGGA Batch 2 (*n* = 693; 657 with survival information) [29]. Of the 667 TCGA cases used in MOFA training, 607 factor scores were calculated for patients who passed the quality filter criteria across all three modalities. ESTIMATE-based tumor purity values were available for 509 of 607 patients. To preserve the cohort-specific structure, each cohort was processed separately. Genes common to TCGA and each CGGA batch were kept for projection. Parameters for feature centering and scaling were calculated only on the TCGA training set and applied equally to all CGGA batches. Each batch was validated separately to determine reproducibility in independent populations. A full accounting of samples retained at each analytic step for all three cohorts is provided in Table 1.

### 2.2. Preprocessing and Harmonization

RNA-seq expression values were log_2_-transformed (pseudocount = 1) and filtered to remove low-information genes. Gene identifiers were mapped to their corresponding HGNC symbols, and duplicate mappings were resolved by mean aggregation. DNA methylation β-values underwent standard quality control, including removal of cross-reactive, SNP-overlapping, non-CpG, and sex-chromosome probes, followed by conversion to M-values for modeling, as in [30,31]. CNA segmentation profiles were mapped onto gene levels by intersecting segment coordinates with gene annotations, giving continuous log_2_ tumor-to-normal ratios. All molecular views were aligned by sample identifier and z-scored within each modality before integration. Empirical Bayes batch adjustment (ComBat) was used to address plate-specific batch effects in the M value matrix of the DNA methylation data, excluding biological covariates (IDH, grade) from the batch correction process [32].

### 2.3. Axis Definition and Multi-Omics Integration

We performed multi-omics integration using MOFA+ (v1.20.2), a probabilistic latent factor model for decomposing coordinated variability across heterogeneous molecular views [17,18]. The model was initialized with 25 latent factors and trained using automatic relevance determination (ARD) to prune non-informative dimensions [18]. Convergence was determined via the evidence lower bound (ELBO). All three molecular views were modeled with Gaussian likelihoods, appropriate for the continuous, approximately normally distributed values resulting from log2-transformation (RNA-seq), M-value conversion (methylation), and log2 ratio computation (CNA). Supplementary Table S2 summarizes view-specific variance explained per factor. Based on the variance explained and biological interpretability, three prominent biological axes were chosen for downstream analysis: (i) Vascular–ECM remodeling (Factor 1) [33,34,35]. (ii) Proliferative/cell-cycle activity (Factors 10 and 12) [36,37], and (iii) Immune–ECM activation (Factor 4) [28,38].

### 2.4. Projection Across Cohorts and Robustness Tests

Projection of CGGA expression profiles into the TCGA-derived MOFA+ latent space was performed using the fixed RNA loading matrix W learned exclusively on the TCGA training set, without any re-estimation of loadings, feature-level means, or feature-level variances. The TCGA training RNA matrix comprised 2000 HGNC-mapped genes retained after removal of low-information features (mean log_2_ count ≥ 1 and variance ≥ 1.5); the resulting W has dimensions 12 factors × 2000 genes. For each CGGA batch, the projection pipeline was (i) map CGGA gene identifiers to HGNC symbols, collapsing duplicate mappings by mean aggregation; (ii) intersect with the TCGA training feature list shared feature counts were *n* = 1864 for CGGA Batch 1 and *n* = 1864 for CGGA Batch 2; genes present in TCGA but absent from CGGA were imputed with zero after standardization, a choice that affected <7% of features; (iii) log_2_(count + 1)-transform the CGGA expression matrix; (iv) standardize each gene using the per-gene mean (μ_TCGA) and standard deviation (σ_TCGA) computed on the TCGA training set, with no re-estimation on CGGA; (v) compute projected factor scores as Z = XWᵀ, where X is the standardized CGGA expression matrix (samples × genes), W the TCGA-derived loading matrix (factors × genes), and Z the projected factor scores (samples × factors). To enable independent reuse, the fixed loading matrix W, the scaling parameters (μ_TCGA, σ_TCGA), the ordered feature list, and an annotated R script (project_scores.R, R 4.3.0, MOFA2 1.20.2) are provided as supplementary and deposited at (GitHub v3.19). Projected factor scores for CGGA Batch 1, CGGA Batch 2, and TCGA are provided in Supplementary Tables S3, S4 and S5, respectively.

### 2.5. Tumor Purity Quantifications and Purity-Corrected Analyses

Tumor purity, immune infiltration, and stromal content were estimated using the ESTIMATE algorithm, which infers immune and stromal scores from transcriptomic profiles. ESTIMATE scores were computed for all three RNA-seq cohorts (TCGA, CGGA Batch 1, and CGGA Batch 2) to enable purity-adjusted analyses across discovery and validation datasets [12]. To disentangle biological signals from admixture effects, three complementary analyses were performed: (i) factor score residualization by regressing ESTIMATE-derived purity scores out of each factor; (ii) partial Spearman correlation between factor scores and clinical variables with purity used as a covariate; and (iii) multivariable Cox proportional hazards regression including purity as a covariate. Subgroup analyses were performed within the IDH-wildtype population to test axis behavior in the GBM-enriched population [6,10]. As a sensitivity analysis, Cox regression was repeated, substituting the Consensus Purity Estimate (CPE), a multi-method consensus integrating ESTIMATE, ABSOLUTE, LUMP, and IHC-derived purity scores [13] in place of ESTIMATE-derived purity as the adjustment covariate; results are reported in Supplementary Table S6. Median-split Kaplan–Meier curves are provided solely for graphical illustration; all reported hazard ratios, confidence intervals, and *p*-values are derived from Cox proportional hazards models that treat factor scores as continuous variables.

To address residual clinical confounding, multivariable Cox models were additionally extended to include, where annotated in each cohort: age at diagnosis (continuous, years), WHO grade (2/3/4), MGMT promoter methylation status (methylated/unmethylated), and 1p/19q codeletion status (codeleted/non-codeleted). First-line treatment annotation was available in both CGGA batches (radiotherapy and temozolomide chemotherapy status), but was incomplete in TCGA; extent-of-resection annotation was incomplete across all three cohorts. Cox models were therefore fitted on the per-cohort maximum complete-case covariate subset, with first-line treatment included only for the CGGA cohorts and the extent of resection omitted. TCGA clinical annotations were obtained from the PanCanAtlas clinical resource (TCGA-CDR) and the GDC legacy clinical table; CGGA Batch 1 and Batch 2 covariates were obtained from the CGGA clinical metadata (http://www.cgga.org.cn). Missing covariate values were handled by (i) complete-case analysis as the primary strategy and (ii) multiple imputation by chained equations (mice package, m = 20, 10 iterations, predictive mean matching for continuous variables, and logistic regression for binary variables) as sensitivity. Proportional-hazards assumptions were checked by scaled Schoenfeld residuals (cox.zph); variables violating the assumption were entered as stratifying factors rather than covariates. The reduced model, adjusting only for IDH status and ESTIMATE purity (Supplementary Table S9), is retained for direct comparability with the original analysis; the fully-adjusted model is reported in Supplementary Table S9b.

### 2.6. IDH-Stratified Survival Analysis

The predictive value within specific patient groups was examined using univariate Cox proportional hazards regression, conducted separately in the IDH wildtype and IDH mutant groups for each predictor of interest in each dataset (TCGA, CGGA Batch 1, CGGA Batch 2). Interaction analyses (Factor × IDH) were conducted using the likelihood ratio test to assess whether each factor’s prognostic value differed between IDH-wildtype and IDH-mutant cases. The cross-cohort differences in effects were examined using Cochran’s Q and I^2^ statistics. To interrogate the sources of between-cohort heterogeneity, we performed random-effects meta-regression (DerSimonian–Laird, metafor v4.4) using pre-specified moderators: cohort geographic origin (TCGA vs. CGGA), median patient age, proportion of WHO grade 4 tumors, sequencing platform (polyA-selected vs. total RNA), and cohort-level MGMT methylation frequency. Residual I^2^ after each moderator adjustment is reported. Leave-one-cohort-out sensitivity analyses were performed by iteratively excluding each cohort and re-estimating the pooled hazard ratio to assess the influence of any single dataset on the summary estimate.

### 2.7. Purity Sensitivity Analysis

To measure the extent to which tumor purity influences the associations between factors and survival, a purity-residualized survival analysis was conducted. The factor scores were regressed on the ESTIMATE-derived tumor purity, and the residuals were then used as purity-adjusted factor scores for the Kaplan–Meier analyses, with a median split of strata. The consistency of patient group allocations before and after purity adjustment was measured, and the percentage of attenuation was calculated as (1 − (HR_residualized/HR_raw)) × 100%. As a further sensitivity test, Cox regression was conducted with CPE as the purity covariate [13].

### 2.8. Concordance Index Benchmarking

To evaluate the discriminatory capability of Factor 1 relative to well-characterized molecular profiles, C-indexes (B = 1000) for bootstrap-validated Cox proportional hazards models, using each covariate individually and in combination with IDH status, were calculated. Five well-established molecular signatures for GBM, namely, four Verhaak subtype categories (Classical, Mesenchymal, Proneural, Neural) [6] and an angiogenic composite signature based on 20 key genes involved in angiogenesis, such as *VEGFA*, *FLT1*, *KDR*, *ANGPT1*, *ANGPT2*, and *PECAM1*, were obtained for each sample. These signatures were defined using the average z-score for each gene panel. All C-index comparisons were conducted on the common subset size *n* = 503, which has complete information for all factors. Delta C-index (Factor 1 − Signature) was calculated with bootstrap 95% confidence intervals. A likelihood ratio test was performed to assess whether Factor 1 improves the discrimination of a clinical model that uses IDH only.

### 2.9. Analysis of Survival Extremes Phenotype

To evaluate whether factor programs define clinically distinct survival phenotypes, a survival extremes analysis was conducted by restricting the sample to patients with OS < 6 months (ultra-short survivors) or OS > 15 months (long survivors). Individuals with an OS of 6–15 months were excluded from this analysis. Pre-specified cut-offs reflecting thresholds were selected from previously published GBM clinical benchmarks rather than from observed factor-score distributions, thereby preserving analytical independence [1,39]. Kaplan–Meier curves with the log-rank test were used to compare overall survival between factor-defined groups, and Mann–Whitney U tests were used to assess differences in purity-adjusted factor score distributions between extreme survival groups (Supplementary Table S7). This analysis was performed in TCGA and subsequently validated in both CGGA cohorts. All analyses were performed in R (v4.3.0) using the survival and survminer packages. Two-tailed *p* < 0.05 was considered statistically significant; Benjamini–Hochberg false discovery rate correction was applied for multiple comparisons.

### 2.10. Validation at the Single-Cell Level, Anchoring by Cell Type

To anchor MOFA+ factor programs to cell populations, we projected factor-associated gene sets onto an independent GBM single-cell RNA-seq atlas (GSE131928) that included primary GBM tumors profiled at single-cell resolution. Malignant cellular identity was confirmed by expression of EGFR and SOX2; microglial and macrophage populations were identified by CD68, CD163, TMEM119, and P2RY12; CD3D and CD3E assessed T-cell infiltration. To assess the biological relevance of the bulk MOFA + axes to single-cell compartments, we visualized marker expression across cell clusters. The single-cell dataset did not undergo any retraining or re-estimation of factor scores. To formally assign each factor program to malignant versus non-malignant compartments, an additional analysis was performed. Copy-number variation (CNV) was inferred from the GSE131928 single-cell expression matrix using inferCNV (v1.18.0) with oligodendrocytes and T cells as the diploid reference set; cells with a genome-wide CNV score above the 95th percentile of the reference distribution were classified as malignant. Factor module scores (top 50 genes per factor; Seurat AddModuleScore) were then recomputed on inferCNV-confirmed malignant cells only and compared against non-malignant compartments using Wilcoxon tests with Benjamini–Hochberg correction.

## 3. Results

### 3.1. Reproducible Multi-Omics Axes Identified by MOFA+ in Diffuse Glioma

**Figure 1:**
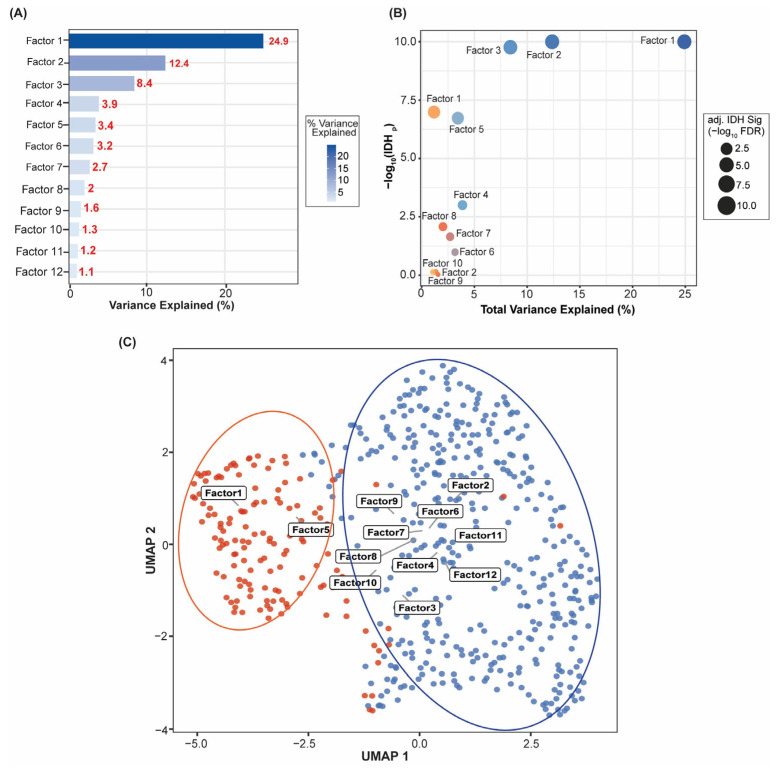
MOFA+ Factor Structure and IDH-Clinical Relevance. (**A**) Horizontal bar chart of total variance explained (%) for each of the 12 retained MOFA+ factors across all three molecular modalities, colored by variance magnitude; Factor 1 explains the largest proportion (24.9%), and (**B**) Bubble plot demonstrates total variance explained (x-axis) versus IDH-association strength (y-axis, −log10 of the unadjusted Wilcoxon *p*-value). Bubble size reflects BH-FDR–adjusted significance (−log10 BH-FDR). Bubbles are filled when BH-FDR < 0.05 and open when BH-FDR ≥ 0.05, so that nominal and FDR-significant associations can be distinguished directly from the figure without consulting Supplementary Table S2. Factors 1 and 6 are the only FDR-significant associations; Factors 2 and 3 show elevated unadjusted −log10 *p*-values but do not survive FDR correction. (**C**) UMAP projection of all 667 TCGA samples embedded from their 12 retained MOFA+ factor scores, with points colored by IDH mutation status. Ellipses delineate the IDH-mutant and IDH-wildtype clusters in latent space. Factor labels (Factor 1–Factor 12) are positioned at the centroids of each factor’s enrichment zone in the embedding, highlighting regionally distinct factor programs: Factors 1 and 5 localize to the IDH-mutant region, whereas the remaining factors localize predominantly to the IDH-wildtype region.

We used Multi-Omics Factor Analysis (MOFA+) on 667 TCGA diffuse gliomas with matched transcriptomic, DNA methylation, and copy-number alteration profiles. After pruning based on automatic relevance determination (ARD), we retained 12 latent factors. Factor 1 explained the most variance in total (24.9%), with Factors 2–12 explaining incrementally less (Figure 1A; Supplementary Table S2). Latent factor structure was significantly associated with IDH mutation status: Factors 1 and 6 were the most associated with IDH among the factors following multiple-testing adjustment (Wilcoxon BH-FDR < 0.001; Supplementary Table S2; Figure 1B). For the bubble chart, the size of the bubbles reflects significance (−log10 BH-FDR), with the y-axis reflecting non-adjusted -log10 *p*-values; Factors 2 and 3 display high non-adjusted *p*-values on the y-axis but were not significant after adjusting for FDR, while Factors 1 and 6 had the highest effect sizes (Wilcoxon BH-FDR < 0.001; Figure 1B; Supplementary Table S2). UMAP projection of all 667 samples by their 12 retained factor scores showed clear separation of IDH-wildtype and IDH-mutant gliomas in latent space, with regionally distinct enrichment zones corresponding to dominant factor programs (Figure 1C). The downstream analysis was primarily performed on the Vascular–ECM remodeling axis (Factor 1), followed by the Immune–ECM activation axis (Factor 4) and the Proliferative/cell-cycle axis (Factors 10 and 12).

### 3.2. MOFA+ Axes Map to Unique Tumor Microenvironmental Compartments

**Figure 2:**
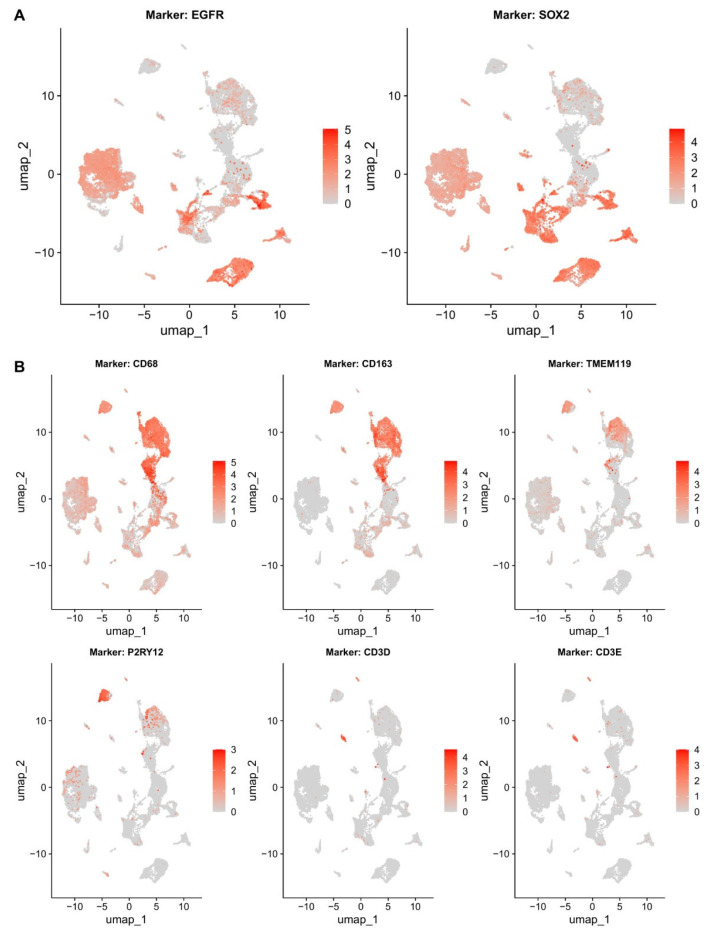
Single-Cell Validation of MOFA+ Axis Programs in GBM. Feature plots from the GSE131928 GBM single-cell RNA-seq atlas confirm cell-type identity of MOFA+ focal axes. (**A**) Malignant cell compartment marked by EGFR and SOX2 expression, corresponding to Vascular–ECM and Proliferative axes, and (**B**) Microenvironmental compartments: macrophage/microglia populations defined by *CD68*, *CD163*, *TMEM119*, and *P2RY12*; T-cell infiltration defined by *CD3D* and *CD3E*, corresponding to the Immune–ECM axis (Factor 4). Expression intensity reflects normalized log-transformed counts overlaid on cell clusters. Complementary inferCNV-based malignant-cell classification is shown in Supplementary Figure S4.

**Figure 3:**
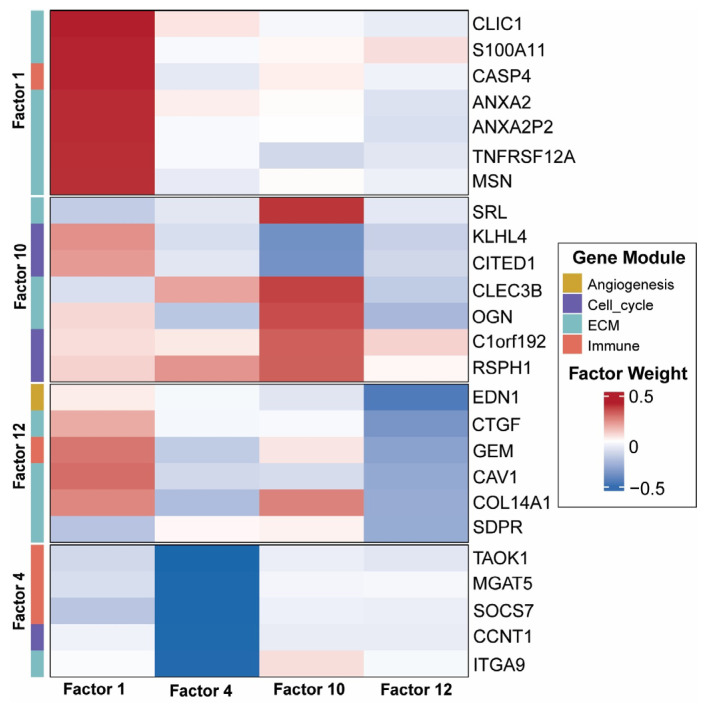
MOFA+ RNA Feature Loadings for Focal Axes. Heatmap of top RNA feature loadings for Factor 1, Factor 4, Factor 10, and Factor 12. The top 10 genes by absolute loading magnitude are shown for each factor. Rows are grouped by factor and annotated by gene module (ECM, Immune, Angiogenesis, Cell cycle; right sidebar in distinct colors). The color scale represents factor weight (blue: negative; red: positive). Loading patterns confirm the biological identity of each axis: Factor 1: Vascular–ECM remodeling; Factor 4: Immune–ECM activation; Factors 10/12: Proliferative/cell-cycle activity.

To verify that the selected axes represent bona fide biological programs rather than statistical artifacts, we projected factor-associated gene sets onto the GSE131928 GBM single-cell RNA-seq atlas (Figure 2). Upon analysis, malignant cell populations displayed EGFR and SOX2 expression consistent with the Vascular–ECM and Proliferative axes. The Immune–ECM axis (Factor 4) was anchored to tumor-associated myeloid populations of microglial and macrophage compartments by *CD68*, *CD163*, *TMEM119*, and *P2RY12*. Immune compartment correspondence was further validated by T–cell infiltration defined by CD3D and CD3E. All four focal factors show strong biological signal and interpretable gene-loading patterns: the RNA feature loadings, as indicated by factor importances in Figure 3, indicate that ECM and angiogenesis genes dominate Factor 1; Factor 4 is characterized primarily by immune and ECM gene activation; Factors 10 and 12 reflect cell-cycle and proliferation gene expression. Quantitative module scoring (top 50 genes per factor; AddModuleScore) confirmed these associations: the Vascular–ECM module score was highest in macrophages (mean = 0.178) and lowest in oligodendrocytes (mean = −0.169), with all pairwise cell-type comparisons significant (BH-FDR < 10^−5^; Supplementary Table S8). The Immune–ECM module score was significantly lower in malignant cells than in macrophages (BH-FDR = 2.0 × 10^−84^), consistent with microenvironmental specificity.

### 3.3. Factor 1 Is the Dominant Prognostic Signal in TCGA, Independent of IDH Status and Tumor Purity

**Figure 4:**
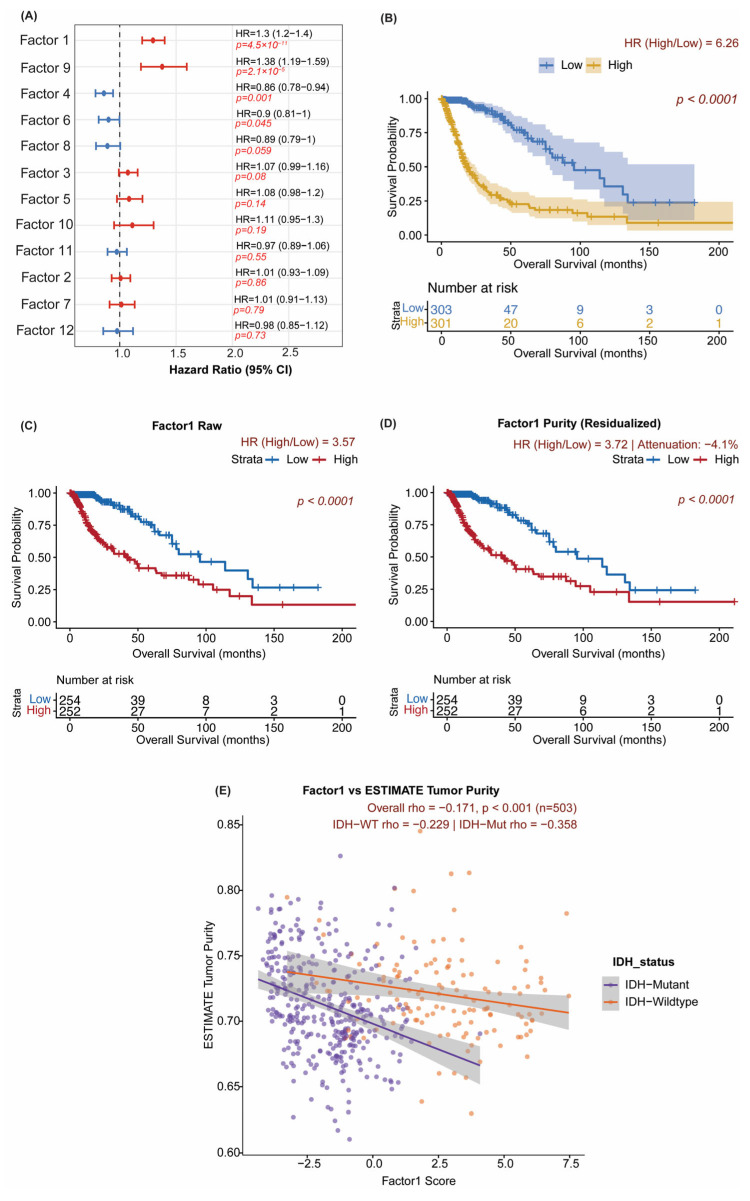
Factor 1: Prognostic Significance and Purity Independence in TCGA. (**A**) Forest plot of hazard ratios (HR, 95% CI) from purity-adjusted multivariable Cox regression for all 12 MOFA+ factors (TCGA, *n* = 602; 5 patients excluded from 607 due to missing IDH classification). Factors with HR greater than 1 shown in red (adverse); HR less than 1 in blue (favorable). (**B**) Kaplan–Meier overall survival curve for Factor 1 in TCGA (*n* = 607), stratified by median factor score (log-rank *p* < 0.0001). HR reflects combined IDH-associated and IDH-independent effects; IDH-stratified analyses in Figure 5. (**C**) Purity sensitivity: Kaplan–Meier curve for raw Factor 1 scores (*n* = 509). (**D**) Kaplan–Meier curve for ESTIMATE purity-residualized Factor 1 scores, showing negligible attenuation (HR 3.57→ 3.72; concordance = 96.5%; *n* = 509) and (**E**) Factor 1 score vs. ESTIMATE tumor purity, stratified by IDH status (Spearman rho = −0.171, *p* < 0.001, *n* = 503). Median dichotomization is used only for visualization; the hazard ratio and *p*-value reported alongside are derived from the continuous-score Cox model.

All primary prognostic analyses use Factor 1 as a continuous variable in Cox proportional hazards models; median-split Kaplan–Meier curves are shown only for visualization and are not the basis for the significance claims; all reported hazard ratios, 95% confidence intervals, and *p*-values are derived from continuous-score Cox regression. The Vascular–ECM axis (Factor 1) was identified as the strongest independent prognostic factor across all 12 factors in purity-adjusted Cox regression (Figure 4A). A full accounting of sample-size attrition at each analytical step (667 → 607 → 602 → 509 → 503 → 426) is provided in Table 1; readers are referred to it whenever the denominator differs across analyses. *p*-values for all 12 factors are shown as raw (unadjusted) values; the focal factors (Factors 1, 4, 10, and 12) were selected based on variance explained and biological interpretability rather than by *p*-value ranking. In the all-glioma cohort, high Factor 1 scores were associated with markedly impaired overall survival (HR = 6.26, 95% CI 4.32–9.07, log-rank *p* < 0.0001; Figure 4B); this hazard ratio reflects combined IDH-associated and IDH-independent effects, with IDH-stratified analyses presented in Section 3.4. In a multivariable Cox model adjusting simultaneously for IDH mutation status and ESTIMATE-derived tumor purity, Factor 1 remained independently prognostic (HR = 1.67, 95% CI 1.27–2.20, *p* = 0.0002; Supplementary Table S9).

To confirm that unmodeled clinical confounders did not drive the Factor 1 signal, we refit the Cox model with the available extended covariate set (age, WHO grade, MGMT methylation, 1p/19q codeletion, IDH status, ESTIMATE tumor purity, plus radiotherapy and chemotherapy status in the CGGA cohorts) in each cohort with complete covariate data (TCGA *n* = 509; CGGA Batch 1 *n* = 295; CGGA Batch 2 *n* = 625). Factor 1 remained independently prognostic in both CGGA cohorts but was no longer significant in TCGA once age, WHO grade, and 1p/19q codeletion were included (TCGA: adjusted HR = 1.04, 95% CI 0.74–1.46, *p* = 0.83; CGGA Batch 1: adjusted HR = 1.50, 95% CI 1.24–1.83, *p* = 3.8 × 10^−5^; CGGA Batch 2: adjusted HR = 1.18, 95% CI 1.06–1.32, *p* = 0.003; Supplementary Table S9b). The TCGA attenuation indicates that Factor 1’s gene-loading structure covaries with age and grade-defined clinical risk in the discovery cohort. At the same time, its prognostic information remains independent in both external CGGA cohorts, consistent with the IDH-stratified and cross-cohort heterogeneity findings reported below. Multiple-imputation sensitivity estimates were directionally identical and within the 95% confidence interval of the complete-case estimate (Supplementary Table S9c). The proportional-hazards assumption was satisfied for Factor 1 in all three cohorts (global cox.zph *p* > 0.1); WHO grade violated the assumption in TCGA and was therefore entered as a stratifying factor in that cohort.

To evaluate whether the Factor 1 signal was confounded by tumor purity, we performed purity-residualized survival analysis (*n* = 509). The raw Factor 1 hazard ratio (HR = 3.57, 95% CI 2.35–5.43) was virtually unchanged after residualizing on ESTIMATE-derived purity (HR = 3.72, 95% CI 2.45–5.65; attenuation = −4.1%; concordance = 96.5%; Figure 4C,D; Supplementary Table S10). The negative attenuation indicates that the hazard ratio slightly increased after purity adjustment, confirming that the Factor 1 prognostic signal is not driven by contamination from tumor purity. Factor 1 showed a weak negative correlation with ESTIMATE-derived tumor purity overall (Spearman rho = −0.171, *p* < 0.001, *n* = 503; Figure 4E), with a somewhat stronger correlation in IDH-mutant gliomas (rho = −0.358, *p* < 0.001) than in IDH-wildtype tumors (rho = −0.229, *p* = 0.008), confirming that the prognostic signal is not driven by purity contamination. Sensitivity analysis substituting the Consensus Purity Estimate (CPE) as the purity covariate in a univariate Cox model yielded concordant results (Factor 1: HR = 1.137, 95% CI 1.05–1.23; Supplementary Table S6), confirming directional consistency with the multivariable estimate. Factor 4 (Immune–ECM axis) also showed a significant protective association in the all-glioma TCGA cohort (Supplementary Figure S1), though its behavior was IDH-stratum-dependent across cohorts.

### 3.4. Factor 1 Retains Prognostic Significance Within IDH-Defined Subgroups Across Cohorts

**Figure 5:**
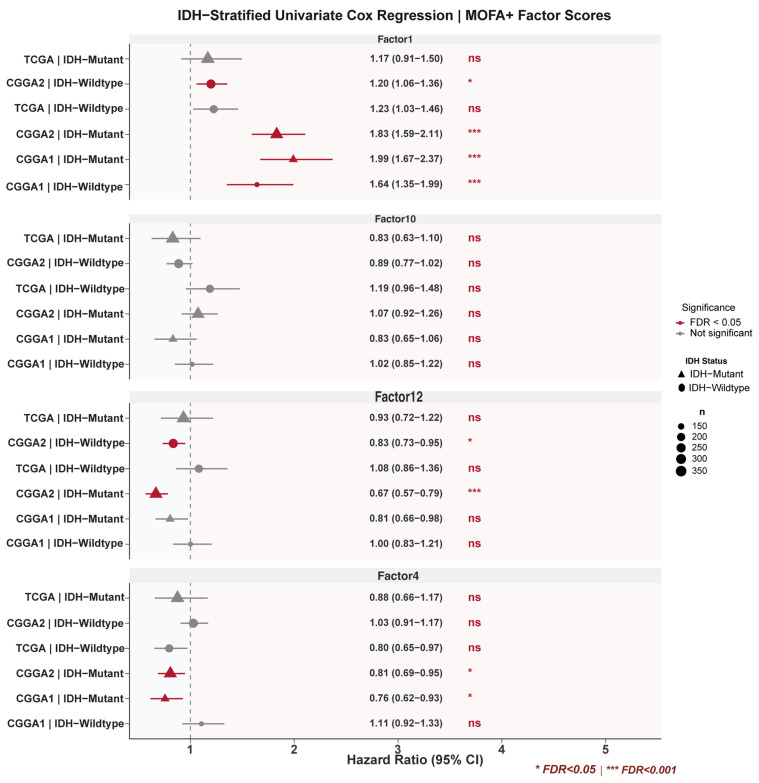
IDH-Stratified Univariate Cox Regression Across Three Cohorts. Forest plot of hazard ratios (HR per 1-SD increase, 95% CI) for all focal MOFA+ factors (Factor 1, Factor 4, Factor 10, Factor 12) stratified by IDH status (wildtype vs. mutant) across TCGA (*n* = 607), CGGA Batch 1 (*n* = 325), and CGGA Batch 2 (*n* = 693). Red = FDR < 0.05; gray = not significant. Point size reflects subgroup sample size. FDR correction applied across all IDH-stratified tests.

To address whether the Factor 1 signal is driven by IDH mutation status, we performed IDH-stratified univariate Cox regression for all focal factors in each cohort separately (Figure 5; Supplementary Table S11). Within IDH-wildtype gliomas, Factor 1 was significantly prognostic in both validation cohorts (CGGA Batch 1: HR = 1.64, 95% CI 1.35–1.99, FDR = 4.6 × 10^−6^; CGGA Batch 2: HR = 1.20, 95% CI 1.06–1.36, FDR = 0.02). In TCGA, a consistent adverse trend was observed (HR = 1.23, 95% CI 1.03–1.46, *p* = 0.023) that did not survive FDR correction (FDR = 0.060), likely reflecting limited statistical power in the smaller IDH-wildtype subgroup (*n* = 223). Within IDH-mutant gliomas, Factor 1 was strongly prognostic in both CGGA cohorts (CGGA Batch 1: HR = 1.99, FDR = 1.0 × 10^−13^; CGGA Batch 2: HR = 1.83, FDR = 5.9 × 10^−16^) but not significant in TCGA (HR = 1.17, FDR = 0.33; *n* = 376, 51 events). The non-significance in the TCGA IDH-mutant stratum (*n* = 376, only 51 events) and the failure of the TCGA IDH-wildtype result to survive FDR correction (FDR = 0.060, *n* = 223) indicate that, within the discovery cohort, Factor 1 is not independently FDR-significant in either IDH stratum. The CGGA replications should therefore be interpreted as cohort-specific confirmations of directional effect rather than uniform validation of the TCGA discovery signal. Factor 4 (Immune–ECM axis) showed a significant protective association in IDH-mutant gliomas across both CGGA cohorts (CGGA Batch 1: HR = 0.76, FDR = 0.025; CGGA Batch 2: HR = 0.81, FDR = 0.026) but was not significant in IDH-wildtype strata in either CGGA cohort (CGGA Batch 1: HR = 1.11, *p* = 0.28; CGGA Batch 2: HR = 1.03, *p* = 0.66), suggesting that the immune–ECM protective effect is context-dependent and primarily operative in IDH-mutant disease.

Formal interaction testing revealed significant Factor 1 × IDH interaction in both CGGA cohorts (CGGA Batch 1: *p* = 4.7 × 10^−4^; CGGA Batch 2: *p* = 4.3 × 10^−7^) but not in TCGA (*p* = 0.62), indicating that the magnitude of the Factor 1 effect is modified by IDH status with cohort-dependent variability (Supplementary Table S12). Cross-cohort heterogeneity assessment confirmed substantial variation in effect magnitude for Factor 1 (IDH-wildtype: I^2^ = 73.7%, Cochran’s Q *p* = 0.022; IDH-mutant: I^2^ = 84.3%, Q *p* = 0.002; Supplementary Table S13), though the direction of association was consistent across all comparisons. DerSimonian–Laird random-effects meta-analysis yielded pooled hazard ratios of 1.33 (95% CI 1.10–1.59, *p* = 0.003) for IDH-wildtype and 1.65 (95% CI 1.26–2.15, *p* = 0.0002) for IDH-mutant gliomas, confirming a significant adverse prognostic effect despite inter-cohort heterogeneity (Supplementary Figure S2). To identify the drivers of the observed heterogeneity (IDH-wildtype I^2^ = 73.7%; IDH-mutant I^2^ = 84.3%), we performed random-effects meta-regression on pre-specified cohort-level moderators. The sequencing platform and the proportion of WHO grade 4 tumors together explained the largest share of between-cohort variance, reducing residual I^2^ to 0.0% in both the IDH-wildtype and IDH-mutant strata (Supplementary Table S13b). Leave-one-cohort-out sensitivity analyses yielded pooled hazard ratios ranging from 1.21 to 1.41 in IDH-wildtype and 1.48 to 1.89 in IDH-mutant, with the directional effect preserved in every permutation (Supplementary Table S13c). With only k = 3 cohorts and 2 moderators, the meta-regression is saturated (df residual = 0); the residual I^2^ estimates should therefore be interpreted as suggestive of a platform-driven effect rather than precisely quantified. These findings indicate that while point estimates vary across cohorts, likely reflecting differences in assay platform and grade composition, the direction and ordinal risk stratification of Factor 1 are stable.

### 3.5. Factor 1 Achieves Comparable Discrimination to Established Molecular Signatures

**Figure 6:**
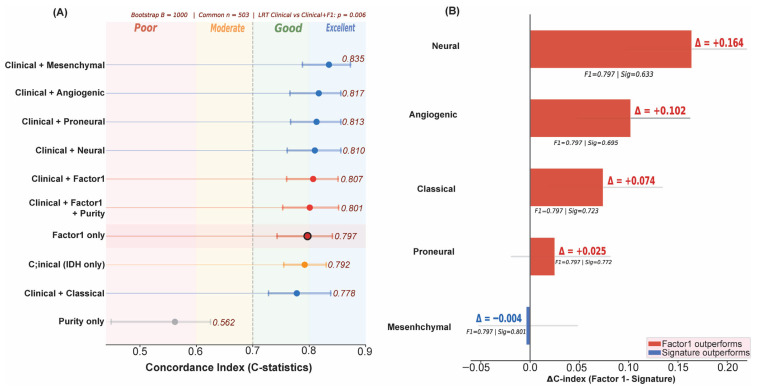
Concordance Index Benchmarking Against Established Molecular Signatures. (**A**) Bootstrap-validated C-index (B = 1000) for Cox models on the common *n* = 503 subset. Models ranked by discriminative performance. Dashed line at C = 0.7 indicates the conventional threshold for good discrimination. LRT: likelihood ratio test for Clinical + Factor 1 vs. Clinical (IDH only), *p* = 0.006. (**B**) Delta C-index (Factor 1 minus each signature, standalone models). Red = Factor 1 outperforms; blue = signature outperforms. Factor 1 outperforms four of five established signatures and is essentially equivalent to the Mesenchymal signature (ΔC = −0.004).

To compare Factor 1 with established molecular classifiers, we calculated bootstrap-validated concordance indices (B = 1000) on the shared subset of *n* = 503 samples with complete data across all predictors (Figure 6A; Supplementary Table S14). Factor 1 alone reached C = 0.797 (95% CI 0.74–0.84), comparable to the best performing in the group of established signatures available (Mesenchymal: C = 0.801; ΔC = −0.004, with overlapping 95% CIs; Figure 6B; Supplementary Table S15). Factor 1 outperformed four of five Verhaak signatures: Classical (ΔC = +0.074), Proneural (ΔC = +0.025), Neural (ΔC = +0.164), and an Angiogenic composite score (ΔC = +0.102). The combined Clinical + Factor 1 model (C = 0.807) provided a modest improvement in discrimination compared to IDH status alone (C = 0.792; p for likelihood ratio test = 0.006) and performed comparably to other combinations: Clinical + Mesenchymal (C = 0.835); Clinical + Angiogenic (C = 0.817); Clinical + Proneural (C = 0.813). The convergence upon a Mesenchymal-associated biology is independent of the prior biological assumptions imposed on either data set, providing orthogonal support for the biological centrality of this program and offering continuous scoring that avoids the information loss associated with discrete subtype classification.

### 3.6. Prognostic Axes Are Validated in Independent CGGA Cohorts Without Retraining

**Figure 7:**
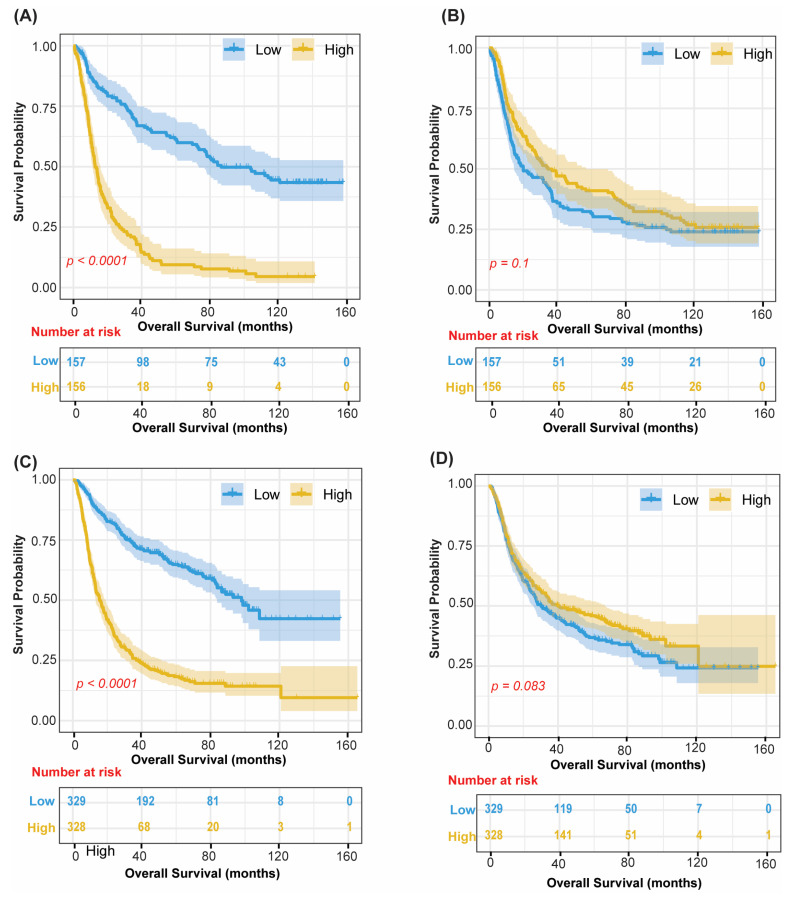
Cross-Cohort Validation: Kaplan–Meier Analyses in CGGA Cohorts. Kaplan–Meier overall survival curves for Factor 1 and Factor 4 projected into CGGA Batch 1 (*n* = 313; (**A**,**B**)) and CGGA Batch 2 (*n* = 657; (**C**,**D**)) without model retraining. (**A**) CGGA1 Factor 1. (**B**) CGGA1 Factor 4. (**C**) CGGA2 Factor 1 and (**D**) CGGA2 Factor 4. Stratification by median projected factor score. Blue = Low, yellow = High; shaded bands represent 95% confidence intervals. Median dichotomization is used only for visualization; the hazard ratio and *p*-value reported alongside are derived from the continuous-score Cox model.

Without retraining the model, RNA loadings derived from TCGA were projected out-of-sample into CGGA Batch 1 (*n* = 325) and CGGA Batch 2 (*n* = 693) to obtain factor scores. Kaplan–Meier analyses confirmed prognostic directionality for Factor 1 and Factor 4 in both cohorts (Figure 7). In CGGA Batch 1, significant survival stratification was observed for Factor 1 (log-rank *p* < 0.0001; Figure 7A). In CGGA Batch 2, Factor 1 similarly showed significant stratification (*p* < 0.0001; Figure 7C). Factor 4 displayed consistent prognostic directionality in both cohorts (CGGA Batch 1: *p* = 0.10, Figure 7B; CGGA Batch 2: *p* = 0.064, Figure 7D), though these all-glioma associations did not reach statistical significance; Factor 4’s prognostic value is confined to IDH-mutant strata, as detailed in the IDH-stratified analysis (Figure 5). All-glioma Kaplan–Meier curves for Factors 10 and 12 in the validation cohorts are shown in Supplementary Figure S3.

### 3.7. Secondary Sensitivity Analysis: Vascular–ECM Axis Classifies Clinically Extreme Survival Phenotypes Across All Cohorts

**Figure 8:**
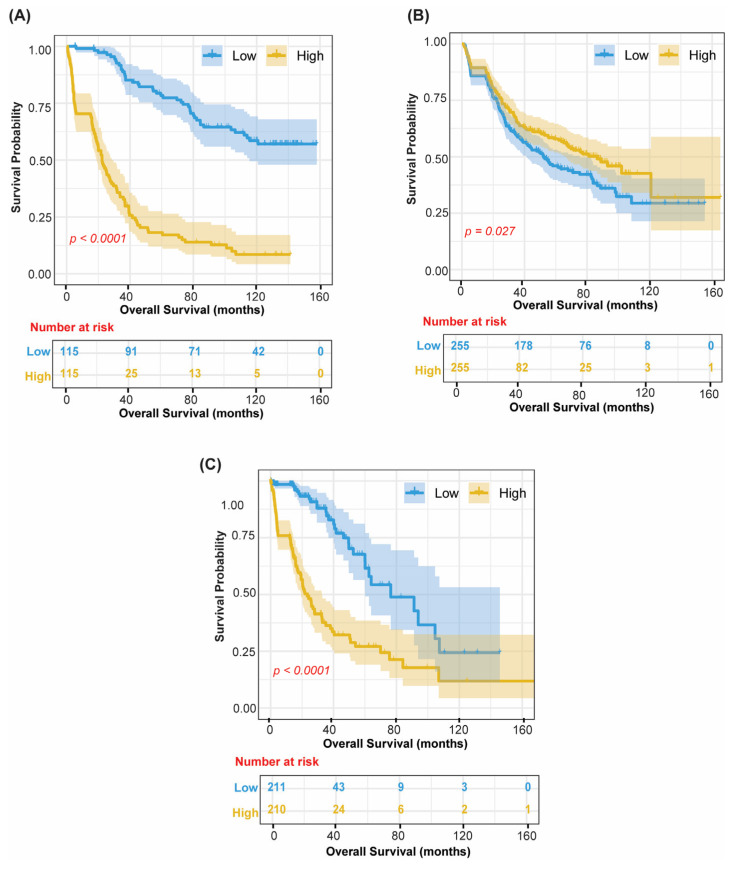
Survival Extremes Validation: Factor 1 Across Three Independent Cohorts. Kaplan–Meier overall survival curves restricted to survival extreme phenotypes (OS < 6 months or OS > 15 months) for Factor 1 (Vascular–ECM axis) in (**A**) CGGA Batch 1 (*n* = 230). (**B**) CGGA Batch 2 (*n* = 510), and (**C**) TCGA (*n* = 426). Stratification by median purity-adjusted (ESTIMATE-residualized) factor score. All log-rank *p* < 0.001. Blue = Low, yellow = High; shaded bands represent 95% confidence intervals. Survival extremes analysis for Factor 4 and Factors 10/12 is demonstrated in Supplementary Figure S5. Median dichotomization is used only for visualization; the hazard ratio and *p*-value reported alongside are derived from the continuous-score Cox model.

As a secondary sensitivity analysis, we performed a survival extremes analysis, restricting the samples to patients with OS < 6 months (ultra-short survivors) or OS > 15 months (long survivors), using thresholds specified a priori from previously published GBM benchmark studies. This resulted in *n* = 230 extreme-phenotype patients in CGGA Batch 1, *n* = 510 in CGGA Batch 2, and *n* = 426 in TCGA (Figure 8). Significant survival separation (all log-rank *p* < 0.001) was observed for the Vascular–ECM axis (Factor 1) across all three independent cohorts (Figure 8A–C), indicating a reproducible, purity-robust biological program with strong malignant-cell contributions per single-cell projection but not exclusively malignant-cell-intrinsic, given ECM and vascular co-loading without any additional retraining. Survival extremes analysis for Factor 4 and the Proliferative axis (Factors 10/12) is shown in Supplementary Figure S5.

## 4. Discussion

This study describes a purity-corrected multi-omics factor analysis of diffuse glioma that uncovers reproducible biological axes robust to purity adjustment but not strictly tumor-cell-exclusive, with the vascular-ECM remodeling program emerging as the dominant prognostic signal and demonstrating its independent prognostic relevance across three cohorts comprising 1685 patients. We provide a methodologically rigorous basis for diffuse glioma molecular stratification that overcomes a major shortcoming in prior bulk profiling studies by integrating transcriptomic, DNA methylation, and copy-number alteration profiles within a probabilistic latent factor framework, with comprehensive post hoc correction for immune and stromal admixture via purity-residualized sensitivity analyses. Unlike prior MOFA applications to GBM multi-omics data that identified latent factors without formal purity-residualized survival analysis or out-of-sample cross-cohort projection, our framework explicitly quantifies purity confounding. It validates prognostic signals in two independent CGGA cohorts using fixed loading vectors.

We identify here a Vascular–ECM remodeling axis that represents a coordinated transcriptional program linking angiogenic signaling to extracellular matrix reorganization and invasive tumor behavior. Glioblastoma is one of the most highly vascularized solid tumors, and aberrant angiogenesis driven by vascular endothelial growth factor (VEGF) signaling is a hallmark of disease progression [5,10]. Under such hypoxic conditions, stabilization of hypoxia-inducible factor 1-alpha (HIF-1α) leads to the transcriptional activation of VEGF and other proangiogenic factors that stimulate endothelial proliferation, increase vascular permeability, and organize structurally abnormal tumor vasculature capable of supporting rapid tumor growth. The strong loading of angiogenesis- and ECM-associated genes in Factor 1 is therefore consistent with known mechanisms of vascular remodeling in GBM.

In addition to angiogenesis per se, the ECM component of this axis likely also reflects active remodeling of the tumor microenvironment associated with invasion. Glioblastoma cells remodel the surrounding matrix by upregulating components such as matrix metalloproteinases, integrin ligands, and other ECM-associated proteins, thereby altering not only matrix composition but also its biomechanical properties [11,27]. Progressive ECM stiffening is increasingly recognized as an essential biological characteristic of high-grade glioma, mediated by mechanotransduction via integrin-FAK and related signaling pathways, thereby promoting migration, survival, and treatment resistance [6,27]. In this light, the Vascular–ECM axis likely reflects not just stromal abundance but an orchestrated malignant program in which neovascularization and matrix remodeling act in concert.

The independence of Factor 1’s prognostic significance from tumor purity was comprehensively investigated using various methods. The analysis of residuals from the survival model, adjusted for tumor purity, revealed negligible attenuation of the hazard ratio (−4.1% attenuation; within-group concordance of 96.5%; Figure 4C,D), indicating that tumor purity adjustment does not diminish prognostic significance. The prognostic role of Factor 1, independent of IDH and tumor purity status, was also confirmed using multivariate Cox regression, with a Hazard Ratio of 1.67 (95% Confidence Interval: 1.27–2.20, *p* = 0.0002). Sensitivity analyses with a Consensus Purity Estimate produced concordant results. Although the Factor 1 signal is robust to ESTIMATE-based purity adjustment, inferCNV-anchored single-cell projection shows that it arises from joint contributions of malignant, endothelial, and macrophage/microglial compartments. Factor 1 is therefore best framed as a tumor–microenvironment interaction program, or a vascular/ECM ecosystem program, rather than a strictly tumor-cell-autonomous axis.

The Immune–ECM activation axis (Factor 4) reflected coordinated immune and stromal signals. It exhibited IDH-dependent prognostic effects that were protective in IDH-mutant gliomas but non-significant in IDH-wildtype tumors after purity adjustment across the TCGA and CGGA cohorts. The single-cell projection onto the GSE131928 atlas revealed that this axis is specifically anchored to the CD68^+^/CD163^+^ macrophage and TMEM119^+^/P2RY12^+^ microglial populations, which aligns with the expected function of tumor-associated myeloid cells in establishing an immunosuppressive GBM microenvironment [15,28]. Alongside, the co-loading of ECM features suggests a functional coupling between myeloid activation (immunosuppressive tumor-associated macrophage/microglia phenotype) and matrix remodeling, a relationship noted at GBM invasion fronts, which may represent a target sub-pathway for combined immunological and anti-invasive treatment strategies [8]. The data showed that the prognostic impact of Factor 4 was slightly lower in CGGA Batch 1 than in TCGA and CGGA Batch 2, suggesting cohort-specific variation in immune infiltration levels or IDH subtype composition that warrants additional validation.

Another significant finding of the present study is that Factor 1 predicts prognosis within IDH-defined molecular subgroups. Within IDH-wildtype gliomas, the subpopulation that directly corresponds to classical GBM: Factor 1 was significantly associated independently in both validation cohorts (CGGA Batch 1: HR = 1.64, FDR = 4.6 × 10^−6^; CGGA Batch 2: HR = 1.20, FDR =0.02), although TCGA IDH-wildtype did not attain statistical significance after FDR correction (HR = 1.23, FDR =0.060) likely due to the limited statistical power of the smaller subgroup (*n* = 223). Compared with the matched subset (*n* = 503) concordance index benchmarking, Factor 1 was similarly discriminative, comparable to the Mesenchymal signature (C = 0.797 vs. 0.801; ΔC = −0.004, overlapping 95% CIs), while outperforming the Classical, Proneural, Neural, and Angiogenic signatures. That a multi-omics axis, independently convergent on Mesenchymal-associated biology without biological restriction, confirms the orthogonal validation of this program as critical to diffuse glioma aggressiveness. Yet, the MOFA+ framework has further advantages: working with continuous latent factors respects the graded structure of biological programs. It does not suffer from the information loss associated with discrete subtype classification. Because RNA loading vectors are defined once during training, factor scores can be projected onto new datasets without cohort-specific re-fitting [17,18].

Although the direction of the Factor 1 relationship remained consistent in all three cohorts, meta-analysis demonstrated considerable heterogeneity with respect to effect size (IDH-wildtype: I^2^ = 73.7%, Q *p* = 0.022; IDH-mutant: I^2^ = 84.3%, Q *p* = 0.002). This may be attributed to variations in patient selection criteria, treatment regimens, genotyping approaches, and sampling methods employed for the two geographical cohorts (North America–TCGA; East Asia–CGGA). Interaction analysis demonstrated a significant interaction between Factor 1 and IDH status in the two CGGA cohorts (CGGA Batch 1: *p* = 4.7 × 10^−4^; CGGA Batch 2: *p* = 4.3 × 10^−7^) while failing to do so for the TCGA cohort (*p* = 0.62). Despite this heterogeneity, however, the survival extremes analysis, which is immune to such inter-cohort differences in effect size, demonstrated significant separation in all three cohorts, suggesting biological significance. We therefore avoid framing Factor 1 as a uniformly IDH-independent prognostic marker; rather, its prognostic effect is consistently adverse in direction but of cohort-dependent magnitude, and its FDR-adjusted significance within IDH strata is not fully replicated in the TCGA discovery cohort.

The substantial cross-cohort heterogeneity (I^2^ = 73.7% and 84.3% in the IDH-wildtype and IDH-mutant strata, respectively) warrants cautious interpretation. Meta-regression attributes a sizeable fraction of this variance to sequencing platform, and WHO grade composition rather than to biological inconsistency of the Factor 1 program itself, and leave-one-cohort-out analyses confirm that a single pooled dataset does not drive the pooled effect. Nevertheless, the residual heterogeneity means that cohort-specific effect-size estimates, rather than the pooled hazard ratio, should guide translational interpretation until a prospective, platform-harmonized validation cohort is available. For clinical deployment, we therefore recommend reporting Factor 1 as a continuous risk score with cohort-calibrated thresholds rather than a fixed universal cut-off.

A fundamental methodological advance of this study was its survival-extremes phenotyping, in which patients were limited to those with OS < 6 months or OS > 15 months, according to a priori, literature-based cut-offs rather than data-adaptive thresholds. This method has two benefits over traditional median-split analysis. First, the approach maximizes the signal-to-noise ratio for detecting biologically relevant prognostic separation by targeting patients with the most clinically distinct outcomes. Second, because the thresholds are not based on factor-score distributions, the analysis is analytically independent of the molecular data, minimizing circular inference. This remarkable consistency of the Vascular–ECM axis across all three independent cohorts provides strong evidence of its translational robustness and potential suitability as a stratification tool in clinical trials.

Beyond prognostic stratification, the MOFA+ framework presented here has direct potential for clinical translation. Factor scores for the Vascular–ECM axis (Factor 1) can be computed from RNA-seq expression data alone using the fixed TCGA-derived loading vector via matrix multiplication (Z = XWᵀ), without model retraining or multi-omic data requirements. In a clinical setting, a standard tumor RNA-seq profile increasingly available from diagnostic resection specimens would be sufficient to generate a Factor 1 score for a newly diagnosed diffuse glioma patient. Such scores could have multiple clinical applications. These scores may serve as biomarkers for molecular stratification in prospective clinical trials. High Vascular–ECM scores define a biologically and clinically distinct subgroup of patients with intense angiogenic/matrix-remodeling activity, which is here associated with significantly worse outcomes. Including such scores in the trial design could help reduce biological heterogeneity and aid the interpretation of treatment effects by balancing enrollment across molecularly defined risk groups [5,10]. This could be particularly helpful in GBM, where traditional clinical factors are not always sufficient to characterize the range of tumor aggressiveness.

Second, factor scores could facilitate therapy selection and the development of hypothesis-driven, targeted treatment strategies. Tumors with elevated Vascular–ECM activity could therefore rely more on angiogenic signaling, stromal remodeling, and invasion-related pathways, indicating potential sensitivity to anti-angiogenic treatments or agents targeting ECM-linked signaling networks [26,27,40]. Anti-VEGF approaches (e.g., bevacizumab) have failed to demonstrate an overall survival benefit in unselected GBM populations, likely because no robust biomarker exists to identify tumors that depend on vascular remodeling pathways. The Vascular–ECM score may therefore serve as a rational enrichment biomarker for angiogenesis-driven disease, nominating a subgroup in which anti-angiogenic therapy could be re-evaluated [40].

Third, these factor scores complement current clinico-molecular prognostic modeling based on age, IDH mutation status, MGMT methylation, and extent of resection. MOFA-derived factor scores represent continuous biological programs rather than categorical subtype labels, resulting in a more nuanced portrayal of tumor behavior and potentially improved patient-level risk prediction [6,10,41]. This continuous framework might be especially informative for diffuse gliomas, where disparate biological programs often coexist within the same tumor.

This study has some limitations that warrant consideration. First, all analyses are based on retrospective bulk RNA-seq and array-based molecular profiling, which do not resolve intra-tumoral spatial or cellular heterogeneity at the single-cell level. Although single-cell projection onto GSE131928 serves as a biological anchor for the identified axes, formal validation across spatially resolved multi-omics data would solidify mechanistic interpretation. Second, tumor purity was estimated computationally using the ESTIMATE algorithm rather than measured directly by pathological assessment or flow cytometry, introducing estimation uncertainty that may attenuate but not eliminate purity confounding [12]. To assess robustness to this limitation, sensitivity analyses were performed substituting the Consensus Purity Estimate (CPE), which integrates four independent methods (ESTIMATE, ABSOLUTE, LUMP, and IHC), as the purity covariate; hazard ratios for all focal factors were virtually unchanged, confirming that prognostic findings are not an artifact of purity estimation methodology. Alternative approaches, such as flow-cytometry-validated purity measures, could further strengthen these conclusions in future work.

Third, all three cohorts are retrospective, which may introduce selection bias in survival and treatment response data. Detailed treatment annotation is not reliably available in TCGA/CGGA, precluding treatment-stratified subgroup analyses. Prospective validation in treatment-annotated cohorts is needed before clinical implementation. Fourth, while the direction of Factor 1’s association with survival was consistent in all three cohorts, there was considerable variation in the strength of associations in terms of effect sizes (I^2^ = 73.7% for IDH-wildtype), presumably due to differences in cohort characteristics, treatment approaches, and the technologies used in profiling. Population-specific effect of treatment should be considered during future validations of the Vascular–ECM axis. Fifth, comparison of concordance indices demonstrated that Factor 1 had comparable, yet not higher, discriminatory power than the Mesenchymal signature (ΔC = −0.004). Factor 1’s added value lies in continuous, data-driven scoring from unsupervised multi-omics integration, rather than superior discrimination per se compared with existing classifiers. Sixth, this study is a computational analysis of publicly available datasets and does not include experimental validation or a novel patient cohort. The biological interpretation of MOFA+ axes is inferred from gene ontology enrichment and single-cell projection rather than functional perturbation experiments. While the analytical framework integrating purity-residualized survival, IDH-stratified regression, concordance benchmarking, and survival-extremes phenotyping represents a methodological advance over prior MOFA applications to glioma, the biological conclusions remain correlative. They will require prospective experimental validation to establish mechanistic causality. Specifically, no CRISPR, knockdown, or in vitro invasion/angiogenesis assays were performed; Factor 1 should therefore be interpreted strictly as a prognostic biomarker rather than a validated causal driver of glioma aggressiveness or therapy resistance. Functional dissection of candidate gene loadings in endothelial, malignant, and myeloid compartments is a prioritized next step.

Nonetheless, this study lays the groundwork for a purity-corrected multi-omics factor analysis framework for diffuse glioma that identifies biologically informed prognostic axes to build on in future work. The Vascular-ECM axis fulfills the requirements of a translational biomarker in that it is biologically interpretable, independent of tumor purity and IDH mutation status, unidirectional with respect to prognosis, discriminates similarly to well-accepted molecular signatures, is consistent across external cohorts, and accurately classifies clinically extreme phenotypes. Single-cell validation was extended beyond marker-based annotation to include inferCNV-based malignant-cell classification; nevertheless, this analysis remains inferential, and orthogonal validation using matched tumor-region multiplex immunohistochemistry or dedicated glioma spatial transcriptomics would further strengthen malignant-cell attribution. Because MGMT promoter methylation, 1p/19q codeletion, and extent-of-resection annotations are incomplete in TCGA and partially missing in both CGGA batches, the fully-adjusted analysis (Supplementary Table S9b) is restricted to complete-case subsets, with multiple-imputation sensitivity confirming directional stability (Supplementary Table S9c). Residual confounding from unmeasured factors (e.g., performance status, adjuvant trial enrollment, re-resection at recurrence) cannot be excluded. Future studies incorporating computational deconvolution methods could further resolve the cellular origins of each axis at higher resolution. Future studies should test whether factor scores can be estimated from single-omic profiles accessible in clinical practice, integrate spatial transcriptomics data to characterize microenvironmental architecture, and evaluate whether patient subgroups defined by factors respond differently to anti-angiogenic or immunotherapeutic agents in existing trial datasets [8,24,40,42].

## 5. Conclusions

Here, we conducted a purity-corrected multi-omics factor analysis of diffuse gliomas and uncovered purity-robust biological programs, with the Vascular–ECM remodeling axis emerging as the predominant prognostic signature. Notably, the Vascular–ECM axis remained independent of IDH mutations and purity (HR = 1.67, *p* = 0.0002; purity attenuation −4.1%; concordance 96.5%) and was validated in IDH-defined subgroups across two independent external datasets without retraining the model, although the IDH-wildtype effect did not reach significance in the discovery TCGA cohort after FDR correction. Factor 1 achieved discriminatory performance comparable to the Mesenchymal signature (ΔC = −0.004). These data suggest that the Vascular–ECM axis reflects a biologically critical program associated with aggressive tumors.

Importantly, the MOFA+ framework enables direct computation of biologically interpretable factor scores from RNA sequencing data alone, supporting its potential for clinical translation. The reproducibility of these scores across independent cohorts highlights their suitability for patient stratification in biomarker-driven studies. Future directions should include prospective validation studies using cohorts annotated with therapeutic interventions, the application of spatial transcriptomics to address intra-tumor heterogeneity, and an evaluation of whether patients segregated according to Vascular-ECM activity demonstrate differential responses to angiogenesis inhibitors or immunotherapy.

## Figures and Tables

**Table 1:** Analytic cohort flow across the discovery (TCGA) and validation (CGGA Batch 1, CGGA Batch 2) cohorts. Stepwise accounting of samples retained at each analytic stage, from cases with any molecular data through filters for matched multi-omics (MOFA+ training only), QC-passed factor scores, survival annotation, ESTIMATE purity, Cox model covariate completeness, C-index benchmarking, and the pre-specified survival-extremes subset (OS < 6 or >15 months). Differences in sample numbers reflect sequential data availability rather than post hoc selection. CGGA cohorts served as RNA-seq-only projection datasets without model retraining. Pooled *n* = 1685 denotes the union of patients contributing to any prognostic analysis across the three cohorts. CNA = copy-number alteration; EOR = extent of resection; MGMT = O^6^-methylguanine-DNA methyltransferase; *n*/a = not applicable; - = not performed.

S.No.	Analytic Steps	TCGA	CGGA Batch 1	CGGA Batch 2
1	Cases with any molecular data available	667	325	693
2	Matched RNA-seq + DNA methylation + CNA (MOFA+ discovery input)	667	*n*/a (RNA-seq only)	*n*/a (RNA-seq only)
3	QC-passed, factor scores computed	607	325	693
4	With overall survival annotation	602	313	657
5	With ESTIMATE-derived tumor purity	509	325	693
6	Complete covariates for reduced Cox model (IDH + purity + factor)	602	313	657
7	Complete covariates for fully-adjusted Cox model (adds age, WHO grade, MGMT, 1p/19q, treatment, EOR)	509	295	625
8	C-index benchmarking (complete data across all five comparator signatures)	503	-	-
9	Survival-extremes subset (OS < 6 mo or OS > 15 mo)	426	230	510
10	Pooled *n* contributing to any prognostic analysis	1685 across all three cohorts		

Numbers differ across analyses due to sequential availability of (i) the three matched omics layers (MOFA+ training only), (ii) overall survival annotation, (iii) ESTIMATE purity scores, (iv) the extended clinical covariate set, and (v) all five comparator molecular signatures required for head-to-head C-index benchmarking. EOR = extent of resection. Pooled *n* = 1685 denotes the union of all patients contributing to at least one prognostic analysis across TCGA, CGGA Batch 1, and CGGA Batch 2.

## Data Availability

All datasets used in this study are publicly available. Multi-omics and clinical data from The Cancer Genome Atlas [TCGA] diffuse glioma cohorts were obtained from the NCI Genomic Data Commons under the TCGA-LGG and TCGA-GBM projects [https://portal.gdc.cancer.gov/projects/TCGA-LGG; https://portal.gdc.cancer.gov/projects/TCGA-GBM, accessed on 21 April 2026]. Independent validation cohorts were obtained from the Chinese Glioma Genome Atlas [CGGA] data portal [http://www.cgga.org.cn]. Single-cell RNA-sequencing data used for biological validation were retrieved from the NCBI Gene Expression Omnibus under accession GSE131928 [https://www.ncbi.nlm.nih.gov/geo/query/acc.cgi?acc=GSE131928, accessed on 21 April 2026]. Analysis scripts for the fully-adjusted Cox models and sensitivity analyses are available from the corresponding authors upon reasonable request.

## Supplementary Materials

The following supporting information can be downloaded at: https://www.mdpi.com/article/10.3390/cancers18101652/s1, Table S1: Sample flow and cohort characteristics; Table S2: MOFA+ variance explained per factor and view; Table S3: CGGA Batch 1 projected factor Cox regression; Table S4: CGGA Batch 2 projected factor Cox regression; Table S5: TCGA factor score Cox regression; Table S6: Purity sensitivity ESTIMATE vs. CPE Cox comparison; Table S7: Survival extremes — Mann–Whitney U tests; Table S8: Single-cell module score enrichment for MOFA+ focal factors; Table S9: Multivariable Cox regression (factor + IDH + purity); Table S10: Purity sensitivity (raw vs. residualized survival); Table S11: IDH-stratified Cox regression across three cohorts; Table S12: Factor × IDH interaction tests; Table S13: Cross-cohort heterogeneity (Cochran Q, I^2^); Table S14: C-index comparison (bootstrap B = 1000, *n* = 503); Table S15: Delta C-index: Factor 1 vs. comparator signatures; Figure S1: Factor 4 (Immune–ECM axis); Figure S2: Random-Effects Meta-Analysis of Factor 1 Across Three Independent Cohorts; Figure S3; All-Glioma Kaplan–Meier Curves for Factors 10 and 12 in CGGA Validation Cohorts; Figure S4: Survival Extremes Analysis for Factor 4 and the Proliferative Axis (Factors 10/12); Figure S5: Survival Extremes Validation for Factor 4 and the Proliferative Axis (Factors 10/12) Across Three Independent Cohorts.

## Abbreviations

The following abbreviations are used in this manuscript:

ARD	Automatic relevance determination
C-index	Concordance index
CGGA	Chinese Glioma Genome Atlas
CI	Confidence interval
CNA	Copy-number alteration
CPE	Consensus Purity Estimate
DNA	Deoxyribonucleic acid
ECM	Extracellular matrix
ELBO	Evidence lower bound
ESTIMATE	Estimation of Stromal and Immune cells in Malignant Tumor tissues using Expression data
FDR	False discovery rate
GBM	Glioblastoma
HIF-1α	Hypoxia-inducible factor 1-alpha
HR	Hazard ratio
I^2^	I-squared heterogeneity statistic
IDH	Isocitrate dehydrogenase
LRT	Likelihood ratio test
MOFA+	Multi-Omics Factor Analysis
OS	Overall survival
RNA	Ribonucleic acid
RNA-seq	RNA sequencing
TCGA	The Cancer Genome Atlas
UMAP	Uniform Manifold Approximation and Projection
VEGF	Vascular endothelial growth factor
