# Supplementary material for: A Vascular–Extracellular Matrix Molecular Program Identifies High-Risk Diffuse Glioma Across Independent Multi-Omics
Source: Cancers (Basel). 2026 May 20;18(10):1652. doi: 10.3390/cancers18101652 (PMC13204189; doi:10.3390/cancers18101652)
Supplement: Supplementary file 1 [file cancers-18-01652-s001.zip › Final_Supplementary_Figures_S1_S5.pdf]

A Vascular–Extracellular Matrix Molecular Program Identifies High-Risk Diffuse Glioma Across Independent Multi-Omics

Shamsa Hilal Saleh [1†], Arshiya Akbar [1†], Fareeha Arshad [1], Saniyah Shaikh [1], Volodymyr Mavrych [1], Olena Bolgova [1], Abrar Barakzai [1], Ahmed Abu-Zaid[1], Mohammed Imran Khan [1,3], Itika Arora [1,2]*, and Ahmed Yaqinuddin [1]*

[1] College of Medicine, Alfaisal University, Riyadh, Saudi Arabia; sanzi@kfshrc.edu.sa (S.H.S.); arshiyaakbar2019@gmail.com (A.A.); farshad@alfaisal.edu (F.A.); sanshaikh@alfaisal.edu (S.S.); vmavrych@alfaisal.edu (V.M.); obolgova@alfaisal.edu (O.B.); abarakzai@alfaisal.edu (A.B.); amabuzaid@alfaisal.edu (A.A.-Z.);
[2] Center for Biotechnology, Khalifa University of Science and Technology, Abu Dhabi, UAE
[3] King Faisal Specialist Hospital and Research Center, Jeddah, Saudi Arabia; mikhan@kfshrc.edu.sa
* Correspondence: itika.arora@ku.ac.ae (I.A.); ayaqinuddin@alfaisal.edu (A.Y.); Tel.: +9666535050381(A.Y.)
† These authors contributed equally to this work.

Supplementary Figures

Supplementary Figure S1

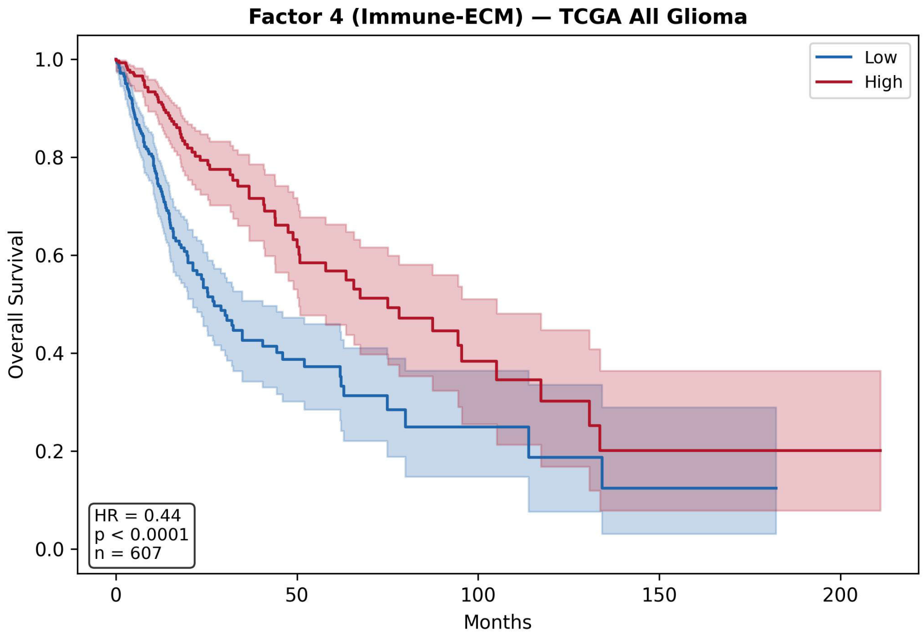

Supplementary Figure S1. Factor 4 (Immune–ECM axis). All-Glioma Kaplan–Meier Survival in TCGA. Kaplan–Meier overall survival curve for Factor 4 in TCGA (n = 607), stratified by median factor score. Factor 4 showed a significant protective association in the all-glioma cohort, though its prognostic value was IDH-stratum-dependent across cohorts. Blue = Low, yellow = High; shaded bands represent 95% confidence intervals.

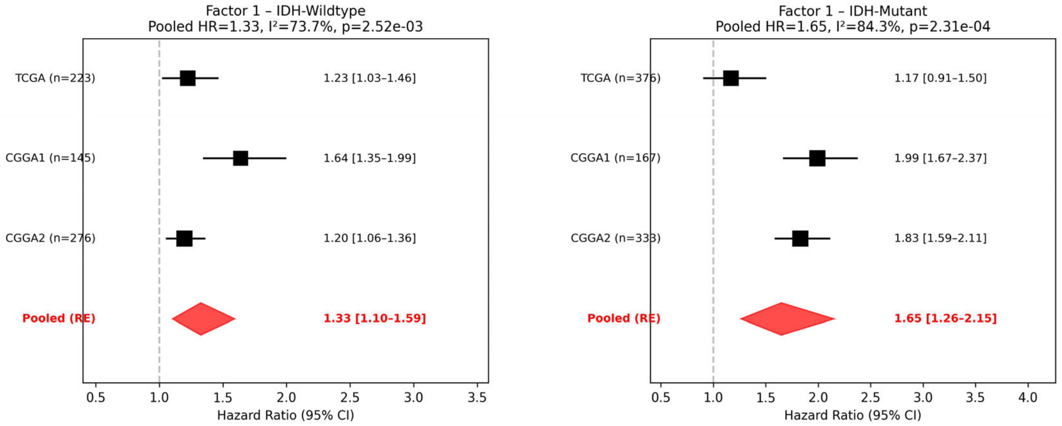

Supplementary Figure S2. Random-Effects Meta-Analysis of Factor 1 Across Three Independent Cohorts. DerSimonian–Laird random-effects meta-analysis of Factor 1 univariate Cox hazard ratios (HR per 1-SD increase) across TCGA, CGGA Batch 1, and CGGA Batch 2, stratified by IDH status. Left panel: IDH-wildtype (pooled HR = 1.33, 95% CI 1.10–1.59, p = 0.003). Right panel: IDH-mutant (pooled HR = 1.65, 95% CI 1.26–2.15, p = 0.0002). Diamonds represent pooled estimates; horizontal lines show 95% confidence intervals for individual cohorts.

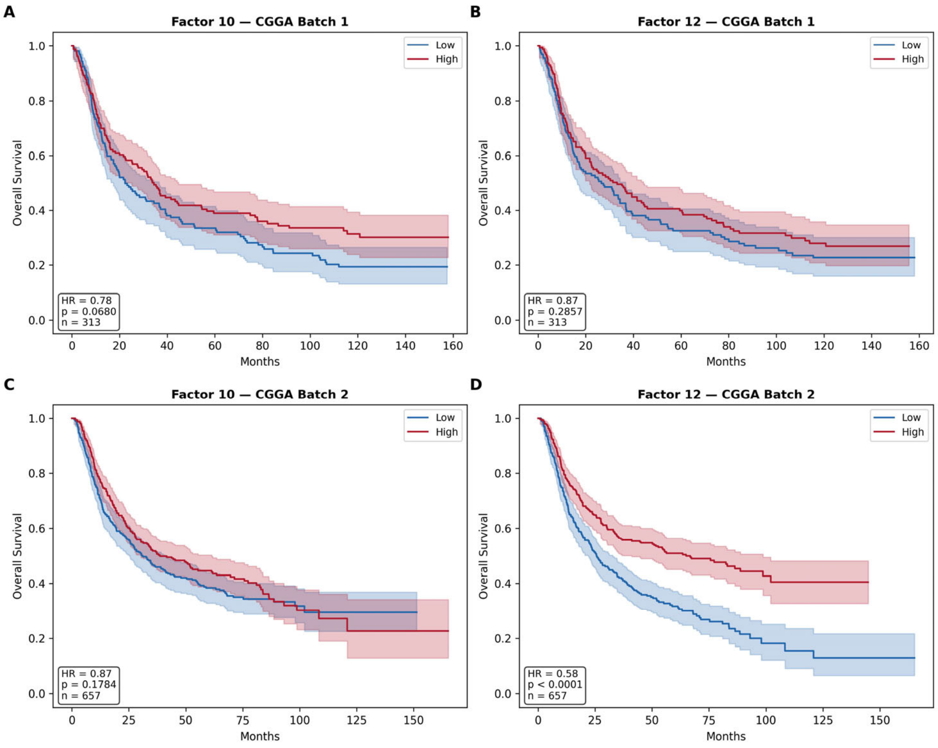

Supplementary Figure S3. All-Glioma Kaplan–Meier Curves for Factors 10 and 12 in CGGA Validation Cohorts. Kaplan–Meier overall survival curves for Factor 10 and Factor 12 (Proliferative/cell-cycle axis) projected into CGGA Batch 1 (n = 313) and CGGA Batch 2 (n = 657) without model retraining. (A) Factor 10 in CGGA Batch 1. (B) Factor 12 in CGGA Batch 1. (C) Factor 10 in CGGA Batch 2, and (D) Factor 12 in CGGA Batch 2. Stratification by median projected factor score. Blue = Low; Red = High; shaded bands represent 95% confidence intervals.

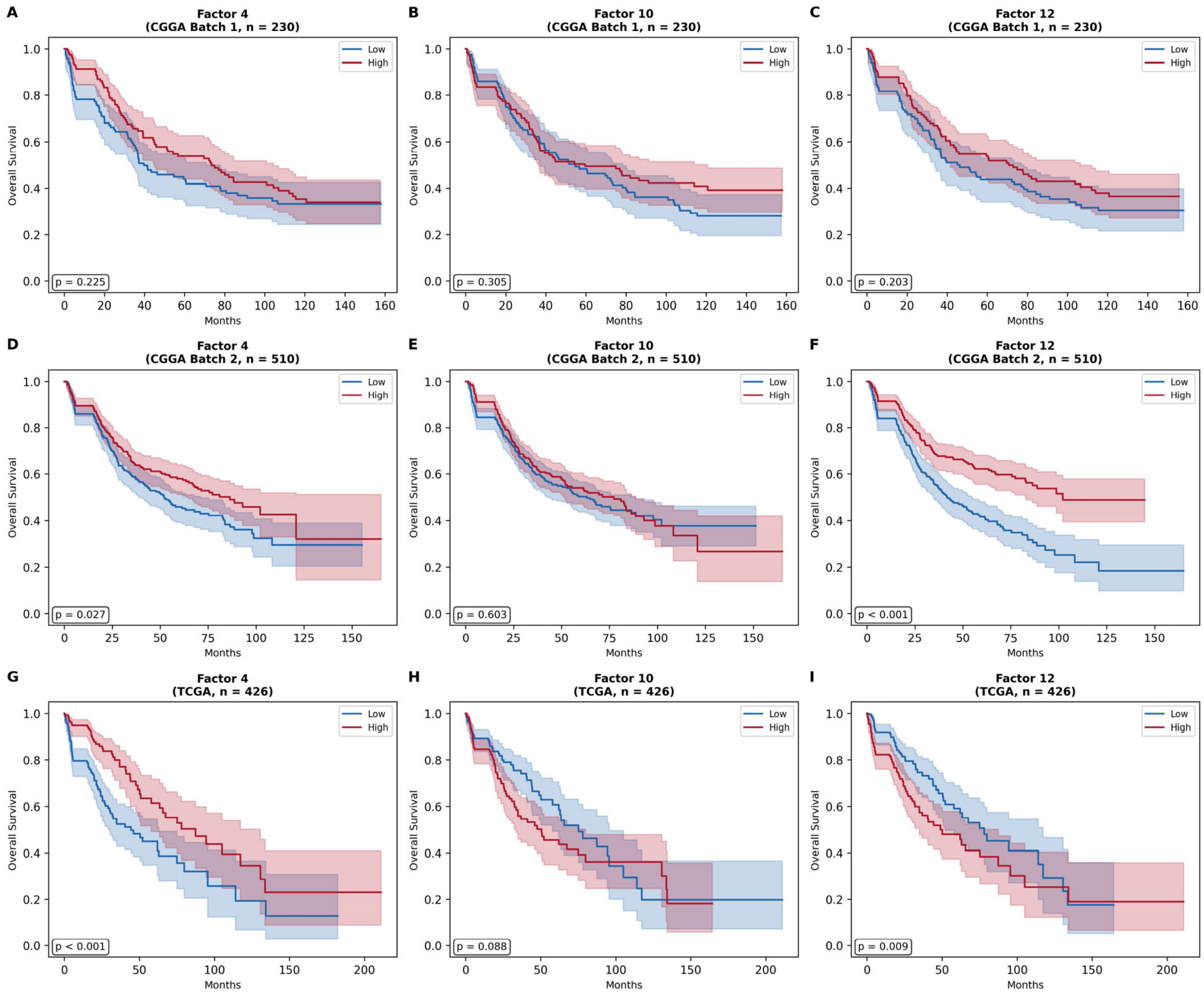

Supplementary Figure S4. Survival Extremes Analysis for Factor 4 and the Proliferative Axis (Factors 10/12). Kaplan–Meier overall survival curves restricted to survival extreme phenotypes (OS < 6 months or OS > 15 months) for Factor 4 (Immune–ECM axis), Factor 10, and Factor 12 (Proliferative/cell-cycle axis) across three independent cohorts. (A–C) CGGA Batch 1. (D–F) CGGA Batch 2 and (G–I) TCGA. Stratification by median purity-adjusted factor score. Blue = Low; Red = High; shaded bands represent 95% confidence intervals.

Supplementary Figure S5. Survival Extremes Validation: Factor 4 and the Proliferative Axis (Factors 10/12) Across Three Independent Cohorts

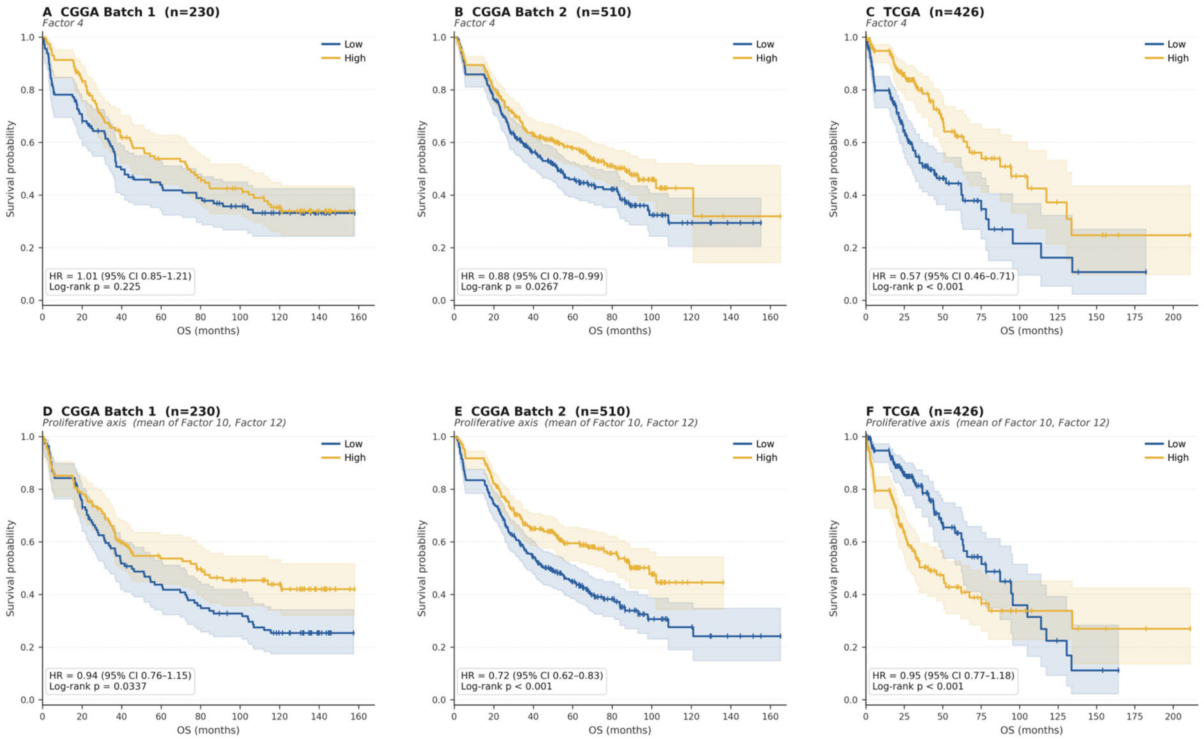

Supplementary Figure S5. Survival Extremes Validation for Factor 4 and the Proliferative Axis (Factors 10/12) Across Three Independent Cohorts. Kaplan–Meier overall survival curves restricted to survival extreme phenotypes [OS < 6 months or OS > 15 months] for Factor 4 in (A) CGGA Batch 1 [n = 230; HR = 1.01, 95% CI 0.85–1.21; log-rank p = 0.23], (B) CGGA Batch 2 [n = 510; HR = 0.88, 95% CI 0.78–0.99; log-rank p = 0.027], and (C) TCGA [n = 426; HR = 0.57, 95% CI 0.46–0.71; log-rank p < 0.001]; and for the Proliferative axis **(**mean of Factor 10 and Factor 12 scores) in (D) CGGA Batch 1 [n = 230; HR = 0.94, 95% CI 0.76–1.15; log-rank p = 0.034], (E) CGGA Batch 2 [n = 510; HR = 0.72, 95% CI 0.62–0.83; log-rank p < 0.001], and (F) TCGA [n = 426; HR = 0.95, 95% CI 0.77–1.18; log-rank p < 0.001]. Stratification by median of the ESTIMATE-residualized factor score in TCGA (the discovery cohort with ESTIMATE purity available) and by median of the raw projected factor score in CGGA Batch 1 and CGGA Batch 2 (out-of-sample projection cohorts without retraining). The Proliferative axis score is defined as the per-sample mean of Factor 10 and Factor 12, reflecting their shared cell-cycle/proliferation programme. Blue = Low, yellow = High; shaded bands represent 95% confidence intervals; ticks denote censoring events. Median dichotomization is used only for visualization; the hazard ratios, 95% confidence intervals, and Cox p-values reported alongside are derived from continuous-score Cox models on the same extreme-phenotype subset (HR per 1 standard deviation of the score).
